# Influence of corn oil recovery on life-cycle greenhouse gas emissions of corn ethanol and corn oil biodiesel

**DOI:** 10.1186/s13068-015-0350-8

**Published:** 2015-11-04

**Authors:** Zhichao Wang, Jennifer B. Dunn, Jeongwoo Han, Michael Q. Wang

**Affiliations:** EcoEngineers, 300 East Locust Street, Des Moines, IA 50309 USA; Systems Assessment Group, Energy System Division, Argonne National Laboratory, 9700 South Cass Avenue, Argonne, IL 60439 USA

**Keywords:** Corn ethanol, Corn oil recovery, Biodiesel, Life cycle analysis, GHG emissions

## Abstract

**Background:**

Corn oil recovery and conversion to biodiesel has been widely adopted at corn ethanol plants recently. The US EPA has projected 2.6 billion liters of biodiesel will be produced from corn oil in 2022. Corn oil biodiesel may qualify for federal renewable identification number (RIN) credits under the Renewable Fuel Standard, as well as for low greenhouse gas (GHG) emission intensity credits under California’s Low Carbon Fuel Standard. Because multiple products [ethanol, biodiesel, and distiller’s grain with solubles (DGS)] are produced from one feedstock (corn), however, a careful co-product treatment approach is required to accurately estimate GHG intensities of both ethanol and corn oil biodiesel and to avoid double counting of benefits associated with corn oil biodiesel production.

**Results:**

This study develops four co-product treatment methods: (1) displacement, (2) marginal, (3) hybrid allocation, and (4) process-level energy allocation. Life-cycle GHG emissions for corn oil biodiesel were more sensitive to the choice of co-product allocation method because significantly less corn oil biodiesel is produced than corn ethanol at a dry mill. Corn ethanol life-cycle GHG emissions with the displacement, marginal, and hybrid allocation approaches are similar (61, 62, and 59 g CO_2_e/MJ, respectively). Although corn ethanol and DGS share upstream farming and conversion burdens in both the hybrid and process-level energy allocation methods, DGS bears a higher burden in the latter because it has lower energy content per selling price as compared to corn ethanol. As a result, with the process-level allocation approach, ethanol’s life-cycle GHG emissions are lower at 46 g CO_2_e/MJ. Corn oil biodiesel life-cycle GHG emissions from the marginal, hybrid allocation, and process-level energy allocation methods were 14, 59, and 45 g CO_2_e/MJ, respectively. Sensitivity analyses were conducted to investigate the influence corn oil yield, soy biodiesel, and defatted DGS displacement credits, and energy consumption for corn oil production and corn oil biodiesel production.

**Conclusions:**

This study’s results demonstrate that co-product treatment methodology strongly influences corn oil biodiesel life-cycle GHG emissions and can affect how this fuel is treated under the Renewable Fuel and Low Carbon Fuel Standards.

## Background

In the past several years, corn oil recovery has been widely adopted in U.S. dry-mill corn ethanol plants, which produce around 90 % of U.S. corn ethanol [1]. Over 80 % of today’s dry-mill ethanol plants have adopted corn oil recovery [2]. One primary use of recovered corn oil is for biodiesel production. In 2014, 440 million kg of corn oil, 10 % of the total mass of biodiesel feedstock, were used for biodiesel production in the United States, while during the same period 2.2 billion kg of soybean oil were used for biodiesel production [3]. The volume of biodiesel produced from corn oil is expected to increase in the future, and the U.S. Environmental Protection Agency (EPA) has projected that 2.6 billion liters of biodiesel could be produced from corn oil recovered from corn ethanol plants in 2022, compared to 2.5 billion liters of biodiesel that could be supported by domestic soy oil production [4]. This volume (2.6 billion liters) is nearly 70 % of the Energy Independence and Security Act mandated level of biomass-based diesel, which was 3.8 billion liters by 2012 [5]. In 2014, 5.4 billion liters of biomass-based diesel was produced. Over 50 % of this volume was produced from soybeans [6].

If it achieves a greater than 50 % reduction in life-cycle greenhouse gas (GHG) emissions compared to conventional diesel, corn oil biodiesel may be eligible to receive renewable identification numbers (RIN) under the Renewable Fuel Standard (RFS2). Moreover, with any level of GHG reductions compared to conventional diesel fuel, corn oil biodiesel could be an eligible biofuel under California’s Low Carbon Fuel Standard (LCFS), which targets a 10 % reduction in the average life-cycle GHG intensity of the ground transportation fuel pool in California by 2020. Eligible fuels can receive LCFS credits in accordance with their carbon intensity (CI) value. Most regulated parties who buy the RIN or LCFS credits for corn oil biodiesel do not trace the origin of the corn oil from which the fuel was produced. As a result, double counting could occur if certain co-product methods are employed in calculating GHG emissions of corn ethanol and corn oil biodiesel or if both the corn ethanol producer that generated the corn oil and the corn oil biodiesel producer claim the credits from corn oil recovery. Under either structure (RFS2 or LCFS) it is important to have a GHG intensity (RFS2) or CI (LCFS) that is calculated with a life cycle analysis (LCA) technique that avoids double counting.

Co-product handling methods applied in biofuel LCA vary widely [7] as there is no consensus on a definitive method and indeed the most appropriate method often varies with the system under consideration. In fact, biofuel-related policies differ on their approach to co-product handling [8]. While the European Union’s Renewable Energy Directive dictates energy allocation, the RFS and LCSF often employ displacement (system expansion) but do not mandate one co-product handling technique. While Ahlgren et al. [9] discuss in detail the different types of co-product handling techniques and their merits and drawbacks, Wang et al. [10] demonstrate the influence of co-product handling techniques on the life-cycle GHG emissions of corn ethanol, soy biodiesel, and cellulosic ethanol. It could be argued that given the influence of co-product handling techniques, sensitivity analysis should be a standard element of biofuel LCAs and several studies do take this approach [e.g., 11–13].

Generally, LCAs of corn ethanol that did not account for corn oil recovery [4, 10, 14, 15] apply the displacement method co-product handling method to the co-produced animal feed [distiller’s grain with solubles (DGS)], which can displace conventional animal feed (i.e., corn, soybean meal and urea). The displacement method allocates all burdens to the main product (i.e., ethanol) and allows the co-products (in this case DGS) to be handled through system expansion. That is, the displacement method considers the influence of the main product, beyond solely its own application, on other systems, in this case, agriculture and animal feed. If applied without caution, however, the displacement method can result in double counting of the GHG emissions reduction credit. For example, no GHG emissions reduction credit should be applied to corn oil biodiesel when corn ethanol claims the credit for displacement of soy-based biodiesel by corn oil biodiesel. Another issue associated with the displacement method’s application to the corn ethanol-corn oil biodiesel system is that it may not allocate the burdens and benefits of corn oil recovery properly because corn oil recovery reduces the DGS drying energy, reducing the DGS energy intensity. This study considers issues that arise in applying various co-product treatment methods to a corn ethanol facility that produces corn ethanol, DGS, and corn oil biodiesel, examining four potential techniques that avoid double counting of GHG emission reductions, and allocates burdens appropriately between the facility’s two fuel products. Further, we explain how these approaches influence CI values of the two fuels and their eligibility under RFS2 and LCFS. This eligibility influences RIN credits biofuel producers receive. These credits, which can at times determine whether biofuels offer a profit margin [16], can influence the biofuels market.

## Results and discussion

**Fig. 1:**
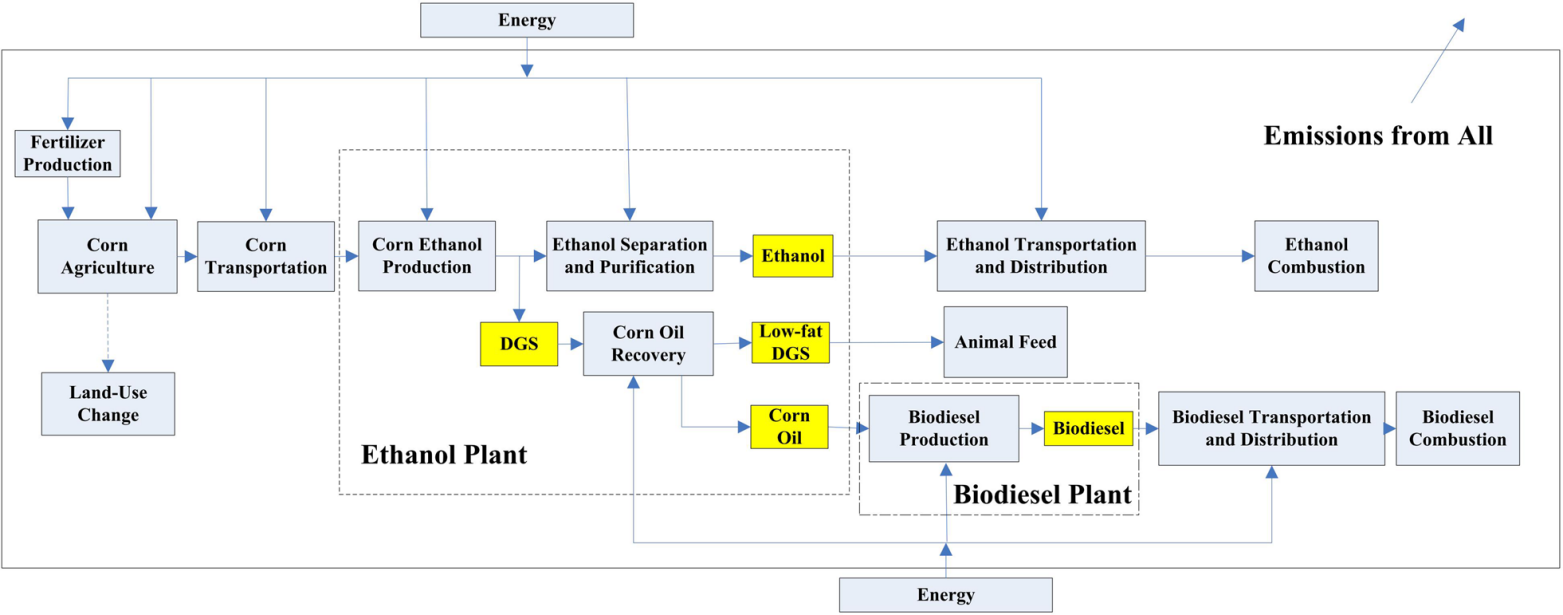
System boundary

In this study, as shown in Fig. 1, the system boundary includes all corn agriculture activities, upstream operations for fertilizer production, transportation of corn to the dry mill, inputs for corn ethanol production, transportation of corn ethanol and biodiesel, and the use of these fuels in vehicles. Domestic and international land-use change associated with corn ethanol is also included, assigned fully to corn ethanol. We later describe how results might change if we allocated LUC between corn oil biodiesel and corn ethanol.

### Co-product handling approaches and key analysis parameters

**Fig. 2:**
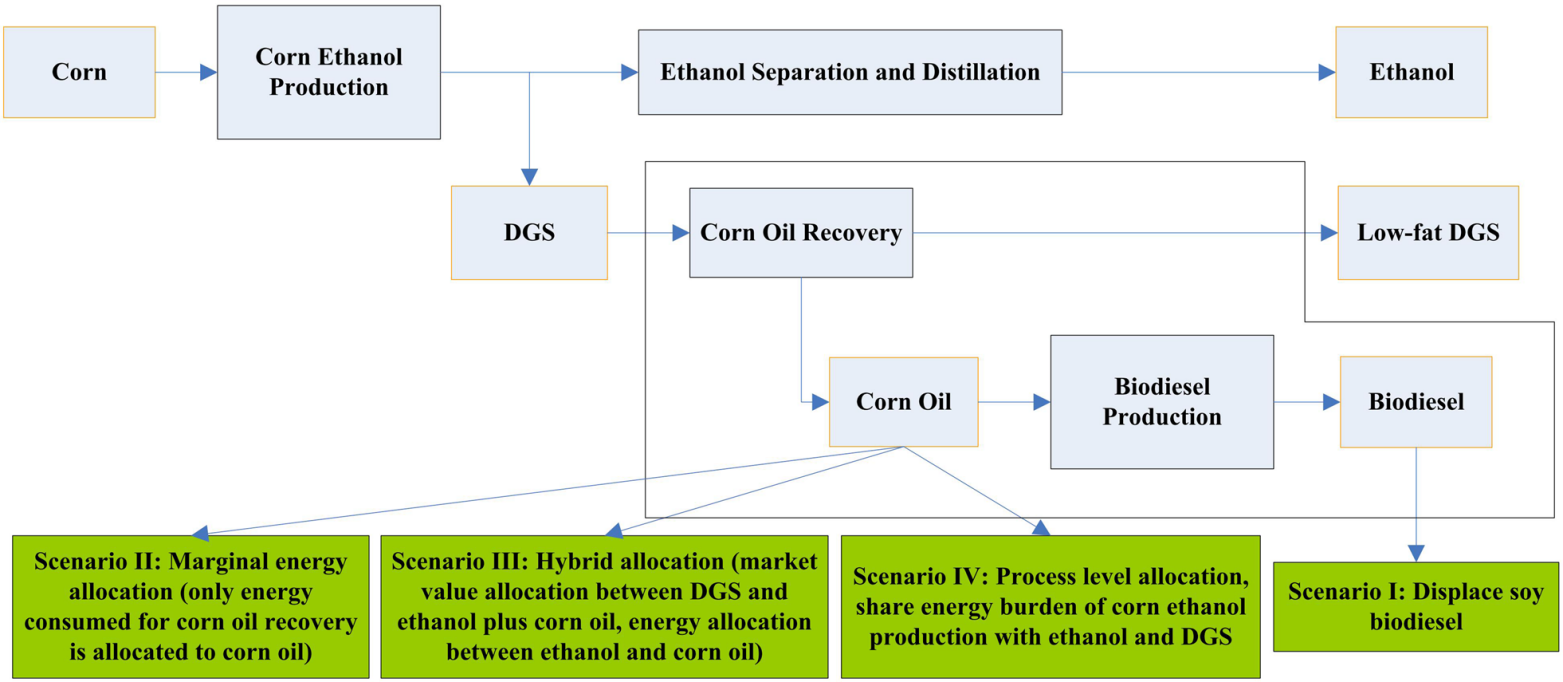
Different treatment methods to handle co-produced corn oil used as a biodiesel feedstock

In this study, we developed four approaches (Fig. 2; Table 1) to investigate how corn oil recovery influences the life-cycle GHG emissions of corn ethanol and corn oil biodiesel with the intention of avoiding double counting of the benefits of co-producing corn oil as a biodiesel feedstock.

**Table 1:** Description of co-product treatment methodologies for each scenario

Process step	I: Displacement	II: Marginal	III: Hybrid allocation	IV: Process-level energy allocation
Corn farming and transport	Allocated to ethanol	Allocated to ethanol	First allocated between DGS and energy products (i.e., ethanol and corn oil) using market value allocation. To calculate individual GHG burdens for the two energy products, energy allocation is used	Allocated to ethanol, DGS and corn oil using energy allocation
Saccharification and fermentation	Allocated to ethanol	Allocated to ethanol		Allocated to ethanol, DGS and corn oil using energy allocation
Ethanol separation and distillation	Allocated to ethanol	Allocated to ethanol		Allocated to ethanol
Corn oil recovery	Allocated to ethanol	Allocated to corn oil		Allocated to biodiesel
DGS drying	Allocated to ethanol	Allocated to ethanol		Allocated to DGS
Biodiesel production	Allocated to ethanol	Allocated to biodiesel	Allocated to biodiesel	Allocated to biodiesel
Displaced DGS credits	Attributed to ethanol	Allocated to ethanol	Not applicable	Not applicable
Displaced biodiesel credits	Attributed to ethanol	Not applicable	Not applicable	Not applicable

These approaches use different co-product handling techniques: (1) displacement, (2) marginal allocation, (3) hybrid allocation, and (4) process-level allocation. The displacement (sometimes called system expansion) technique allocates all production burdens to the main product (ethanol) and applies a credit to ethanol for the co-product’s displacement of a conventional product. For example, a credit for corn oil biodiesel’s displacement of soy oil biodiesel is applied to ethanol. Applying this credit requires a value for the GHG intensity of soy oil biodiesel. We apply a CI for soy oil biodiesel based on an analysis we describe elsewhere [17]. Similarly, corn ethanol receives a displacement credit for the DGS co-product. DGS displaces animal feed at the ratios discussed later in this section.

The second approach, marginal allocation, reflects the perspective that the corn ethanol plant exists to produce corn ethanol; corn oil recovery is a marginal operation. In this approach, corn ethanol bears all the burden of energy consumption during ethanol production except for the energy consumed for corn oil recovery. The energy and GHG intensity of corn oil biodiesel in this approach reflect only the corn oil recovery, biodiesel production process, and transportation and combustion of the final biodiesel product. Similar to the displacement approach, the DGS co-product displacement credit is applied to corn ethanol.

The third approach is called hybrid allocation. The burdens for corn farming and material and energy consumption within the ethanol plant are first allocated between DGS and the combined energy products (i.e., corn oil, ethanol) of the corn ethanol plant using market value allocation. The 3-year moving average market values of corn oil, corn ethanol, and DGS are $0.88, $0.85, and $0.17/kg, respectively [18, 19]. Then to allocate burdens between ethanol and corn oil, energy allocation is used. The reason for this hybrid approach is that DGS is not typically regarded as an energy product while ethanol and corn oil are. Using the market value allocation first may put them onto a relatively fair basis for comparison.

The fourth approach, called process-level allocation, calculates the energy intensity of each step in the biorefinery, including saccharification, fermentation, ethanol separation, ethanol purification, thin stillage evaporation, DGS drying, and biodiesel production [20]. Then, the energy and emission burdens of individual process steps within the system boundary are assigned to the product that is responsible for the existence of the process step. If the step applies to all co-products, they share the burden based on mass or energy content. For example, corn ethanol was assigned all energy consumed in ethanol purification. Energy consumed in corn oil recovery was assigned solely to corn oil. Similarly, only DGS was assigned the energy consumed in DGS drying. We applied energy allocation to divide energy consumed in other upstream processes (e.g., fermentation) that applied to all three co-products. Likewise, the corn agriculture burdens are split between corn ethanol, DGS, and corn oil based on energy allocation.

The energy demand for the unit operations used in each process step was based on a USDA dry-mill ethanol plant process model built in Superpro [20]. Technology advancements, however, have decreased energy consumption at dry-mill corn ethanol plants [1, 14] over time and the USDA process model is nearly a decade old. To address this issue, the energy demand for each unit operation was scaled based on corn ethanol plant energy consumption data [21] recently gathered in 2012. Those authors surveyed 50 % of dry-mill corn ethanol plants and characterized energy consumption at corn ethanol facilities, reflecting the adoption of advanced technologies that save energy. Key parameters for GREET modeling, which produces LCA results (see “Methods”), of corn agriculture, corn ethanol production, and corn oil biodiesel production are presented in Table 2.

**Table 2:** Summary of key parameters

Parameter	Value	Unit	Data source
Corn agriculture			
Corn farming energy	0.40	MJ/kg corn	[27]
Fertilizer usage			
N	17	g/kg corn	[27]
P_2_O_5_	5.7	g/kg corn	[27]
K_2_O	5.9	g/kg corn	[27]
Ethanol production			
Ethanol yield	0.42	L/kg corn	[13]
DGS yield (bone-dry)	0.27	kg/kg corn	[13]
Corn oil yield	0.01	kg/kg corn	[13]
Total energy consumption	7.4	MJ/L EtOH	[13]
Natural gas	6.7	MJ/L EtOH	[13]
Electricity	0.20	kwh/L	[13]
Energy for corn oil recovery	14	kJ/L EtOH	[13, 20]
Energy for distillation and purification	1.1	MJ/L EtOH	[13, 20]
Energy for DGS drying	2.1	MJ/L EtOH	[13, 20]
DGS displacement ratio			
Corn	0.75	kg/kg	[22]
Soybean meal	0.32	kg/kg	[22]
Urea	0.02	kg/kg	[22]
Corn oil biodiesel production			
Corn oil biodiesel yield	0.96	kg/kg	[36]
Corn oil biodiesel energy consumption			
Natural gas	0.90	MJ/kg biodiesel	[36]
Electricity	0.04	kWh/kg biodiesel	[36]

One concern regarding corn oil recovery is that it could influence DGS’s performance as an animal feed. If this were the case, GREET’s existing treatment of DGS displacement of conventional animal feed would need to be modified. GREET contains displacement ratios of 1 kg DDGS for corn, soybean meal (SBM), and urea of 0.751, 0.320, and 0.024 kg, respectively. The total displacement ratio is, therefore, 1.095:1 [22]. The literature suggests that the displacement ratios of DDGS for corn and soybean meals in various livestock diets could range from 1:1 [23–25] to as high as 1.2:1 in beef cattle diets [26]. As we described elsewhere [27], we examined the literature [e.g., 28–30] to determine whether we needed to modify these ratios if corn oil is extracted from DGS. Overall, the literature did not indicate a clear trend in displacement ratio changes when low-fat DGS is used rather than higher fat DGS and that this topic requires further research. In the absence of experimental data that clarify the influence of corn-oil removal on DGS performance, we turned to information about the cost of DGS because price differences between low-fat and higher fat DGS could indicate a performance difference. We looked at DGS price data since corn oil recovery has increased in 2008 and did not observe a clear response in DGS prices to increases in corn oil recovery at dry-mill ethanol plants [31]. In fact, Shurson [31] found that no distinction is made between the two types of DGS based on marketing grades or standards. In the absence of literature or market data that would enable us to revise the default DGS conventional feed displacement ratios in GREET, we have left them unaltered. We did, however, consider the DGS yield reduction of the resulting from corn oil recovery in our analysis.

### Life-cycle GHG emissions

**Fig. 3:**
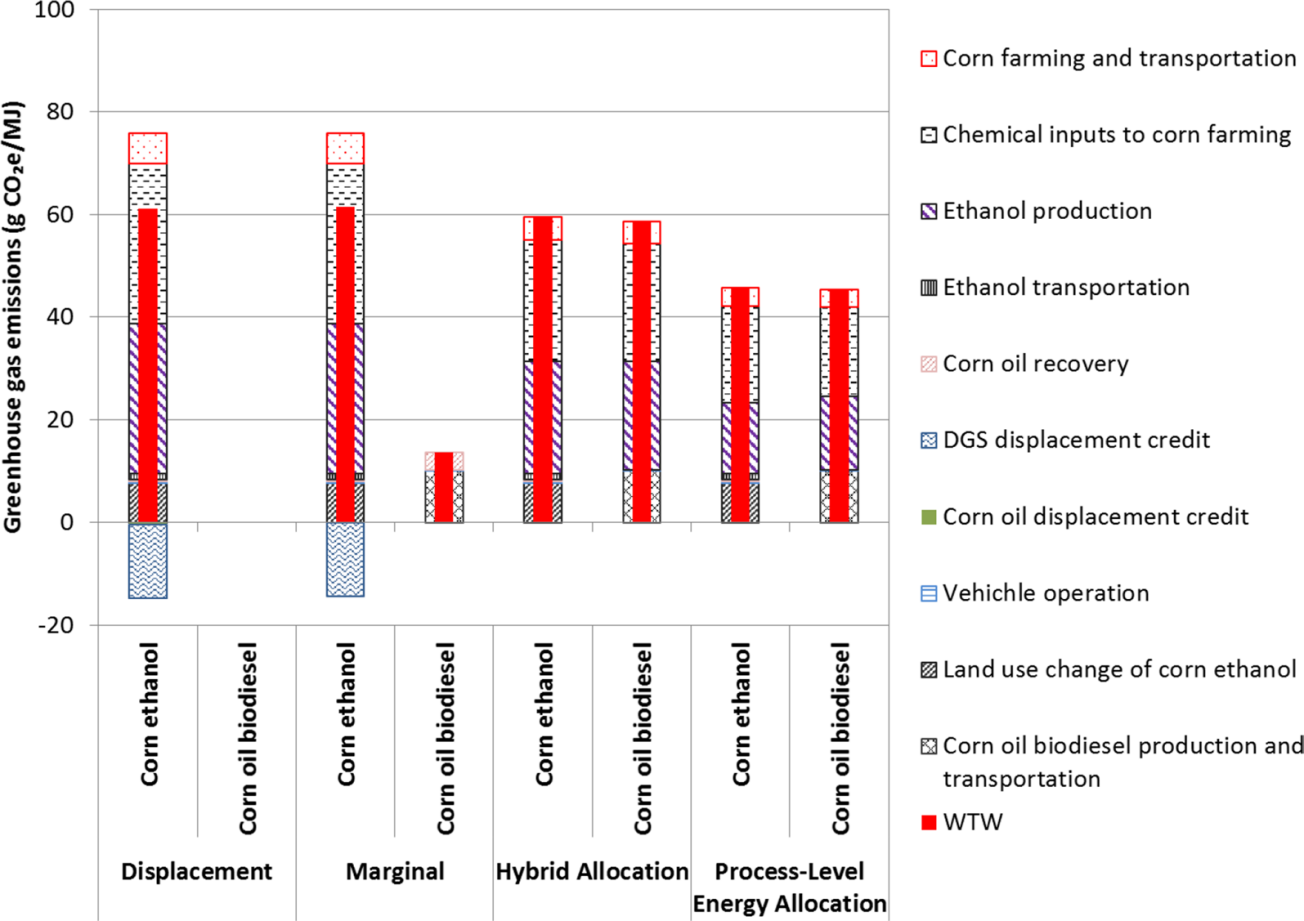
WTW GHG emissions of corn ethanol and corn oil biodiesel in different approaches

Figure 3 shows corn ethanol and corn oil biodiesel life-cycle GHG emissions with the four different approaches to co-product allocation. The displacement approach only provides a CI value for corn ethanol because this fuel receives the displacement credit for corn oil biodiesel (0.4 g CO_2_e/MJ ethanol). The displacement and the marginal approaches produce similar corn ethanol CI results for two reasons. First, the mass of corn oil produced is small compared to that of the mass of corn ethanol and DGS (1 kg corn oil per 35 kg corn ethanol and 30 kg DGS produced). Second, corn oil recovery represents only 0.2 % of total energy consumption at dry-mill corn ethanol plants. Another important point about both of these methods is that they handle DGS through displacement; DGS does not carry a GHG burden itself. The hybrid allocation approach produces a similar corn ethanol CI to the displacement and marginal approaches despite the application of completely different co-product allocation methods. In this approach, DGS has a relatively low GHG intensity (0.47 g CO_2_e/g or 23 g CO_2_e/MJ) because it has a low market value on a per energy-content basis. The process-level energy allocation approach, however, produces a corn ethanol CI that is significantly lower than the other approaches produce. The difference between the hybrid approach and the process-level approach lies in how much of the GHG emissions associated with upstream processing steps (especially ethanol production) are allocated between ethanol and DGS rather than to corn oil. In this approach, DGS bears a higher GHG burden (0.99 g CO_2_e/g or 49 g CO_2_e/MJ) than in the hybrid allocation approach. In the hybrid allocation approach, 76 and 21 % of overall GHG emissions are allocated to ethanol and DGS, respectively. On the other hand, DGS assumes a higher share of the GHG burden (37 %) when the energy allocation method is used. This shift reflects DGS’s lower energy content per selling price as compared to corn ethanol. This shift is a key underlying reason why GHG emissions of both corn ethanol and corn oil biodiesel are lower with the process-level approach than with the hybrid approach.

The influence of co-product handling approaches on corn oil biodiesel CI results is more striking than for corn ethanol CI results. In the marginal approach, corn oil bears only the burden from corn oil recovery, and the CI of corn oil biodiesel is significantly lower (13.5 g CO_2_e/MJ) compared to the hybrid and process-level allocation approaches (59.4 and 45.4 g CO_2_e/MJ, respectively), in which corn oil biodiesel bears a share of upstream GHG emissions. With the displacement method, corn oil biodiesel is burden-free, although it should not receive RIN credits, because its GHG reductions are fully credited in corn ethanol GHG emissions.

The CI values we have generated for corn oil biodiesel can be compared with those estimated by the California Air Resources Board (CARB). CARB has used the marginal approach to estimate the CI for corn oil biodiesel produced from ethanol plants when DGS is dried in 2011 [32], and released another report estimating the CI for corn oil biodiesel produced when DGS is not dried in 2014 [33]. In their 2011 analysis of corn oil produced along with dry DGS, CARB, considering corn oil to be a secondary product to ethanol, assigned only the marginal energy consumed for corn oil recovery to corn oil. At the same time, CARB reduced the co-product credit available to corn ethanol from production of DGS because of the marginal loss in DGS mass and assigned these emissions to corn oil biodiesel. Finally, the marginal energy savings in the DGS drying step (less DGS to dry and improved heat transfer) are also credited to corn oil biodiesel. With this approach, CARB reported [32] a corn oil biodiesel CI value of 4 g CO_2_e/MJ, which is similar to the result we obtained with the marginal approach.

In 2014, CARB [33] published a CI for corn oil biodiesel when DGS is not dried (29.3 g CO_2_e/MJ). In this case, there is no energy savings during DGS drying as a result of corn oil recovery. Again, the reduction in the DGS credit is attributed to corn oil biodiesel. An alternative to this approach that CARB did not consider is to assign this effective penalty to corn ethanol.

From the above comparison, we observe that each of these co-product treatment methods has their own merits and drawbacks. For example, with the displacement approach, corn oil biodiesel cannot play a role in meeting LCFS GHG reduction targets because its GHG reductions are fully credited to corn ethanol. The hybrid and process-level energy allocation approaches have the advantage of more equitable treatment of the two fuel products but one could argue that the full burden of the conversion process should go to corn ethanol, the fuel the conversion facility was built to produce. Finally, in the marginal approach, the corn oil biodiesel has significantly lower WTW GHG emissions largely because the corn farming and transport burdens are not allocated to corn oil. Some may argue that corn oil should also bare a portion of these burdens when corn oil production becomes prominent. Given its influence on CI results and potential policy implications, as biofuel LCA practitioners select a co-product treatment methodology, it is important that they specify this choice transparently and explain its advantages and disadvantages. At the same time, whether the volume of corn ethanol and corn oil biodiesel can be considered in RFS2 and LCFS depends on the co-product method used in LCA.

In all approaches, ethanol production and chemical inputs to corn farming are the most significant contributors to corn ethanol life-cycle GHG emissions, followed by LUC GHG emissions, corn farming, and ethanol transportation. Corn oil recovery contributes minimally to these emissions. For corn oil biodiesel, in the marginal approach, corn oil recovery, biodiesel production and transportation emit about 13.5 g CO_2_e/MJ. But in the hybrid and process-level energy allocation approaches when corn oil shares upstream burdens with corn ethanol and DGS, these steps become the third largest contributor to life-cycle GHG emissions after chemical inputs to corn farming and ethanol production.

### Sensitivity analysis

Over the course of our analysis, we identified five key parameters related to corn oil recovery that could significantly influence life-cycle GHG emissions of corn ethanol and corn oil biodiesel (Table 3). We varied these parameters by −25 to 25 % in a sensitivity analysis to assess this influence. Some of these parameters are unlikely to change by as much as 25 %. For example, the corn oil displacement ratio for displacing soy oil is unlikely to reach 1.25. Nonetheless, we altered each parameter by the same amount to test results’ sensitivity to these parameters.

**Table 3:** Key parameters for sensitivity analysis

Parameter	Nominal	Low	High	Unit
Corn oil yield	0.01	0.008	0.013	kg/kg corn
DGS displacement ratio	1.1	0.82	1.36	kg/kg
Corn oil displacement ratio	1	0.75	1.25	kg/kg
Energy consumption for corn oil recovery	14	11	18	kJ/L EtOH
Corn oil biodiesel energy consumption	0.99	0.75	1.24	MJ/kg biodiesel

**Fig. 4:**
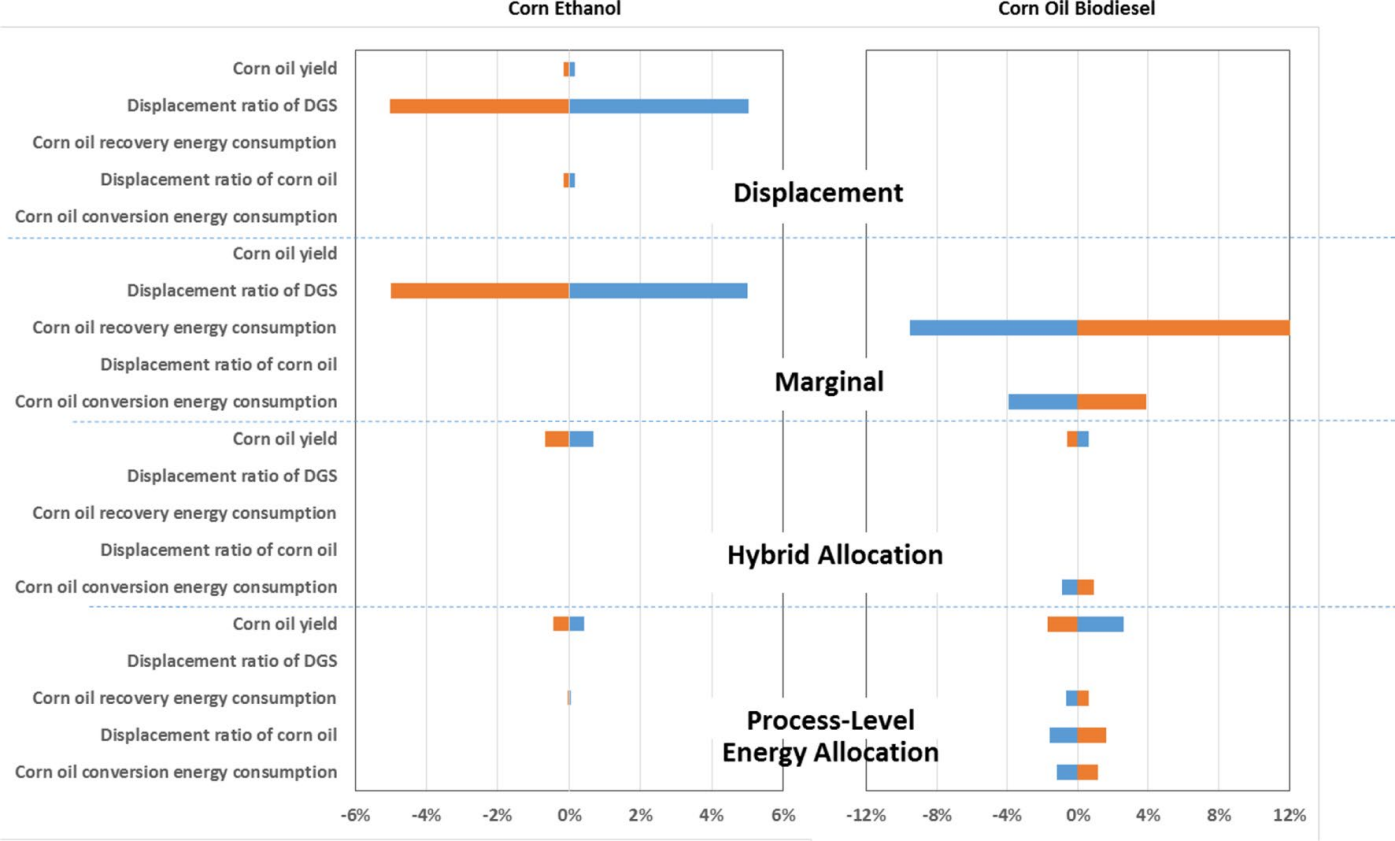
Sensitivity analysis of WTW GHG emissions of corn ethanol and corn oil biodiesel

Figure 4 shows the sensitivity analysis results. Overall, in all approaches, varying the parameters in Table 3 by ±25 % did not change the WTW GHG emissions of either corn ethanol or corn oil biodiesel more than 5 %, with the exception of corn oil recovery energy consumption in the marginal approach. For corn ethanol, in both displacement and marginal approaches, the DGS displacement ratio has the greatest influence on WTW GHG emissions. For corn oil biodiesel, in the marginal approach, corn oil recovery energy consumption has the greatest influence, while in the hybrid and process-level energy allocation approaches, no parameters have influences greater than 4 %. These results show that for this system (Fig. 1), the co-product treatment method influences WTW GHG emissions of corn ethanol and corn oil biodiesel more than the parameters’ values.

### Other important issues

In this study, we did not consider how land-use change (LUC) GHG emissions would change when corn ethanol plants recover corn oil. We assigned all LUC GHG emissions to corn ethanol based on the conditions and assumptions used in the modeling that led to our estimate of corn ethanol LUC GHG emissions [34]. If we had applied energy allocation to assign LUC GHG emissions to both corn ethanol and corn oil, however, those emissions would be 7.3 g CO_2_e/MJ, only 0.3 g CO_2_e/MJ lower than the value for corn ethanol with no allocation. This minor change is a result of the low volume of corn oil biodiesel produced as compared to corn ethanol. Notably, GHG emissions for corn oil biodiesel in the hybrid and process-level energy allocation approaches would be 7.3 g CO_2_e/MJ higher. It is important to note, however, that a full analysis of corn oil biodiesel LUC GHG emissions that takes into account the interplay between corn oil biodiesel, corn ethanol, and soy biodiesel would likely produce different results.

Taheripour and Tyner [35] demonstrate that corn oil recovery and conversion to biodiesel does influence LUC GHG emissions for both corn ethanol and soybean biodiesel. In their analysis, the recovery and conversion of corn oil to biodiesel reduces the LUC GHG emissions associated with producing both corn ethanol and soy biodiesel and, therefore, generates a credit. These authors analyzed four approaches in which the credit is allocated differently. These four approaches are (1) assign the credit to ethanol industry; (2) assign the credit to the biodiesel industry; (3) share the credit between corn ethanol and biodiesel industry using energy allocation; and (4) assign the credit to corn oil biodiesel, which has no associated LUC GHG emissions. Under the different approaches, the change in induced LUC emissions for corn ethanol caused by corn oil recovery is all less than 1 g CO_2_e/MJ. In contrast, the reduction of LUC GHG emissions for biodiesel could be as high as 7 g CO_2_e/MJ biodiesel. Tapping corn oil as a biodiesel feedstock could, therefore, notably reduce overall biodiesel LUC GHG emissions.

Another important issue is how different treatments of corn oil recovery in corn ethanol and biodiesel LCA may influence the RIN and LCFS credits the biofuel producers may obtain. The RIN credit a biofuel receives is based on the combination of feedstock, fuel and process that produce it. Once this combination is defined, life-cycle GHG emissions are calculated, and the appropriate biofuel category (conventional, renewable, advanced, cellulosic) is established. Corn ethanol is considered a conventional biofuel and its production is capped at 15 billion gallons per year.) Each biofuel category has its own RIN (Table 4). For example, if ethanol is produced from corn using fermentation and achieves at least a 20 % GHG emissions reduction compared to gasoline, it will be assigned a D6 RIN. On the other hand, corn oil biodiesel produced using transesterficiation qualifies for D4 RINs which have a minimum of 50 % GHG reduction. It is possible that some co-product treatment techniques (e.g., the process-level allocation approach), along with energy and material efficiency improvements in the corn ethanol supply chain, could render corn ethanol as having a 50 % reduction in life-cycle GHG emissions as compared to gasoline. The RFS, however, restricts corn ethanol to the conventional biofuel category and this fuel can only qualify for D6 RINs. In the marginal and process-level allocation approaches we explored, as well as in the two CARB studies, corn oil biodiesel has more than 50 % GHG reductions compared to its counterpart from petroleum. With the displacement method corn oil biodiesel cannot generate RINs.

**Table 4:** RIN category and their GHG reduction threshold

RIN category	D-code	GHG reduction threshold (%)
Cellulosic biofuel	D3	60
Biomass-based diesel	D4	50
Advanced biofuel	D5	50
Renewable fuel	D6	20

On the other hand, the value of a biofuel under the LCFS is based on its CI value, which means the monetary value of the credit is calculated on the basis of g CO_2_e reduction per MJ fuel compared to baseline petroleum fuels. No emissions reductions thresholds exist. Therefore, the LCFS credit is more sensitive to the actual CI value, or GHG reduction, of a biofuel. As our analysis demonstrates, the CI value of corn oil biodiesel is more sensitive to co-product treatment methodology than corn ethanol. Corn oil biodiesel may have very different CI values depending on which co-product treatment method is used and how the DGS credit reduction is allocated. This variation in CI value has a direct effect on biofuel prices in the LCFS structure and could significantly affect the biodiesel industry. It should be noted that, when corn oil biodiesel and corn ethanol are produced at the same facility, as the CI value for corn oil biodiesel fluctuates given the co-product handling technique, the corn ethanol CI value must be adjusted accordingly under the LCFS constructs.

## Conclusions

Incentives for biofuel production can lead to opportunities for intentional or unintentional double claiming of credits, be they tax, volume production, or GHG emissions reductions, by multiple parties. Recently, three individuals pleaded guilty in a federal case that prosecuted them for purchasing biodiesel for which tax credits had already been claimed, reselling the fuel, and claiming the tax credits anew [36]. In the case of CI values for co-produced biofuels, transparent and traceable accounting of CI value development and accounting for produced fuel volumes accordingly are essential such that multiple fuels do not receive credit for the same GHG reduction effect.

This study examined the influence of four co-product treatment methods on life-cycle GHG emissions of corn ethanol and corn oil biodiesel when corn oil is recovered from corn ethanol production. Each method, together with appropriate fuel volume accounting, avoids double counting of the benefit of displacing soy biodiesel by corn oil biodiesel. The results demonstrate that for corn oil biodiesel, co-product treatment methodology has a significant influence on life-cycle GHG emissions which in turn affects how this fuel is handled under two key biofuel policies—the RFS and LCFS. This influence is important for regulatory agencies and the biofuels community to consider because policy and regulation can sway biofuel markets and production.

## Methods

This study uses an LCA framework to investigate the life-cycle GHG emissions of corn ethanol and corn oil biodiesel production. To calculate these emissions, we employed the GREET^TM^ (Greenhouse gases, Regulated Emissions, and Energy use in Transportation) model developed at Argonne National Laboratory. GREET^TM^ is publicly available and investigates the life-cycle energy use, greenhouse gas emissions, water consumption, and air pollutant emissions of various vehicle technologies and transportation fuels [37].

## Abbreviations

CARB: California Air Resources Board
CI: carbon intensity
DGS: distiller’s grain with solubles
EPA: environmental protection agency
GHG: greenhouse gas
GREET: greenhouse gases, regulated emissions, and energy use in transportation
LCA: life cycle analysis
LCFS: Low Carbon Fuel Standard
LUC: land-use change
RFS2: Renewable Fuel Standard
RIN: renewable identification number
US: United States
